# Effects of chronic exposure to thiamethoxam on larvae of the hoverfly *Eristalis tenax* (Diptera, Syrphidae)

**DOI:** 10.7717/peerj.4258

**Published:** 2018-01-17

**Authors:** Kate Basley, Balin Davenport, Kate Vogiatzis, Dave Goulson

**Affiliations:** 1 School of Life Sciences, University of Sussex, Brighton, East Sussex, UK; 2 Department of Life Sciences, Imperial College London, London, UK

**Keywords:** Pesticide, Chronic exposure, Mortality, Non-target, Weight, Syrphidae, Neonicotinoid, Larvae

## Abstract

There is widespread concern over the use of neonicotinoid pesticides in the agro-ecosystem, due in part to their high water solubility which can lead to widespread contamination of non-target areas including standing surface water. Most studies investigating the negative fitness consequences of neonicotinoids have focused on bees, with little research on the impact on other non-target insects. Here we examined the effect of exposure on the aquatic larval stages of the hoverfly *Eristalis tenax* L. (Diptera: Syrphidae) to a range of concentrations (control, 5, 15, 50, 100 and 500 ppb) of the neonicotinoid thiamethoxam; no published studies have thus far examined the effects of neonicotinoids on hoverflies. Survival was significantly lower when exposed to 500 ppb thiamethoxam, but this concentration exceeds that likely to be found in the field. We observed no effect on survival, development or any latent effects on adult activity budgets resulting from exposure to lower concentrations (up to 100 ppb). Our results suggest that *E. tenax* exposed as larvae to thiamethoxam are unlikely to be negatively impacted by this neonicotinoid under field conditions.

## Introduction

Beneficial insects play an essential role in the functioning of natural ecosystems and pollination is perhaps the best documented of the ecosystem services provided by insects (Vanbergen & Insect Pollinators Initiative, 2013). The economic value provided by wild pollinators is on par with that provided by managed honeybees (Kleijn et al., 2015), which is approximately one-third of all pollination service demands in the UK (Breeze et al., 2011). It is therefore vital to understand the causes behind the reported widespread population declines of many pollinators (Biesmeijer et al., 2006; Carvalheiro et al., 2013; Potts et al., 2010; Burkle, Martin & Knight, 2013; Jauker et al., 2012).

In many countries, land use is dominated by agriculture which has been subject to major change due to the industrialisation of food production and the advent of increased mechanisation and chemical-input (Robinson & Sutherland, 2002). Neonicotinoid pesticides, first introduced to the global market in the mid-1990’s (Jeschke et al., 2011), have been rapidly adopted and are now used in over 120 different countries, on hundreds of different crops, via soil drenches, sprays and most commonly, as seed dressings (Morrissey et al., 2015). When applied as a seed treatment, 1–2% of the active ingredient is released onto the wind as dust (Tapparo et al., 2012). Some of the active ingredient is subsequently taken up by the plant, however owing to neonicotinoids high water solubility, on average about 90% is lost to the soil (Goulson, 2013). This can lead to widespread contamination of farms and the surrounding environment, with potential for impact on both pollinators and predatory insects (Botías et al., 2015, 2016; Krupke et al., 2012; Rundlöf et al., 2015; Jones, Harrington & Turnbull, 2014).

Surface waters, including puddled water, ditches, irrigation channels and streams in and near farmland have been found to be contaminated by neonicotinoids (Morrissey et al., 2015; Van Dijk, Van Staalduinen & Van der Sluijs, 2013; Samson-Robert et al., 2014; Main et al., 2014; Schaafsma et al., 2015). For example, thiamethoxam, one of the most commonly used pesticides from the neonicotinoid group (Simon-Delso et al., 2015), has a relatively long half-life in soil and high water solubility (average DT_50_ = 229 days, 4,100 mg/L) which means it is persistent in the environment with high potential to be transported into surface water via run-off or groundwater discharge (Main et al., 2014). A recent survey of water monitoring literature focussing on surface water contamination by neonicotinoids, found thiamethoxam levels to range from 0.001 to 225 ppb (Morrissey et al., 2015). Even low levels of neonicotinoids have been associated with negative effects on aquatic invertebrates, evident at both the individual and population level (Pisa et al., 2015); for example, the LC_50_ for imidacloprid and the mayfly *Ceriodaphnia dubia* is 2.1 ppb (Chen et al., 2010).

Neonicotinoid pesticides act as agonists of the nicotinic acetylcholine receptors, resulting in excitation, paralysis and death of the target insect (Moens, De Clercq & Tirry, 2011). Numerous studies have raised concerns over the use of neonicotinoid pesticides and the risks to bees, suggesting that exposure to field-relevant doses can impair pollen collection, increase worker mortality, reduce the production of new queens, weaken the bee’s immune system and affect the weight of honeybee queens (Gill & Raine, 2014; Gill, Ramos-Rodriguez & Raine, 2012; Whitehorn et al., 2012; Di Prisco et al., 2013; Gajger, Sakač & Gregorc, 2017). However, little research has focused on other non-target insects.

Hoverflies (Syrphidae) are often considered to be the second most important pollinators after bees (Larson, Kevan & Inouye, 2001). Evidence suggests *Eristalis tenax* (Linnaeus) has pollination value in open and closed crop production systems, and at high densities has a pollination effort comparable to the efficacy of small honeybee colonies (Jauker et al., 2012). Some species of hoverfly are also valued biocontrol agents since their larvae eat aphids (Ramsden et al., 2016). Additionally, approximately half of all hoverflies have saprophagous larvae (Gilbert et al., 1994), these species play an essential part in the decomposition and recycling process of a wide variety of materials, including compost, dung and dead wood, by breaking up and aerating the substrate as they move through it (Gilbert, 1985). Therefore, it is prudent to encourage hoverfly populations on farmland to maintain a healthy functioning ecosystem, at a time where other pollinators like bees are suffering serious declines due to a wide range of stressors (Goulson et al., 2015). In addition, we need to ascertain if there are any latent sublethal effects on adult function stemming from larval exposure which may impair their value as pollinators.

The repeated application of insecticides can lead to a significant loss of dipteran larvae and a potential accumulation of dead organic material in surface water (Sanchez-Bayo, 2011); however, there is a dearth of studies investigating the impact of neonicotinoids on the aquatic larvae of Diptera (Pisa et al., 2015). The authors are aware of no published studies that have investigated the impact of neonicotinoids on Syrphidae and, due to the inherent differences in physiology among species, considerably more research is required (Pisa et al., 2015). Here, we experimentally test the effect of field-realistic doses of a commonly used and highly persistent neonicotinoid, thiamethoxam, on the development of the aquatic larvae, and latent effects in adult behaviour, of the hoverfly *E. tenax.*

## Method

### Study organism and rearing method

Female *E. tenax* deposit eggs on the surface of stagnant water or decaying material and, under laboratory conditions, eggs hatch within two to three days (K. Basley, 2016, personal observation). The aquatic larvae filter-feed on microbes in decaying organic matter, and respire using an extended anal segment used as a breathing tube (Rotheray, 1993). Once fully grown, larvae exit the aquatic habitat in search of a dry shaded place in which to pupate. Adults feed on both pollen and nectar and, in the UK, can be found on the wing from late March to early December (Ball & Morris, 2013).

To produce a suitable silage substrate for oviposition, two weeks before the beginning of the experiment, three 14 L buckets were filled with a mixture of grass clippings and water. Fresh grass clippings were from the University of Sussex campus where there is no history of neonicotinoid usage. Three more buckets were created using a larch (*Larix decidua)* sawdust and water mix. Buckets were covered in a very fine insect proof muslin, to prevent any insects from ovipositing in the mixture. All six buckets were left outside to allow to decompose for two weeks. The grass clippings were then strained through muslin to produce ‘grass silage,’ and the collected water, designated ‘silage water,’ was retained. The sawdust buckets were also strained, the sawdust solids were retained but the water was discarded. The grass silage and sawdust solids were further squeezed to remove excess water and used in varying ratios to produce, an oviposition tray substrate or to create either a holding lagoon or neonicotinoid-treated experimental lagoon substrate.

To obtain larvae of a known age, prospecting female *E. tenax* were collected from a large heap of grass clippings on the University of Sussex campus (50°52′N, 0°4′W) between May and August 2016, one week before the start of each experimental round. Females were returned to the laboratory and placed inside mesh cages (60 cm × 45 cm × 60 cm) under UV light and provided with pollen, 15% sucrose solution w/v, and mineral water (ASDA, own brand). A tray (30 cm × 40 cm × 6 cm) filled with a 2:3 mixture (by weight) of grass silage and ‘silage water’ (see above for preparation) with dried leaves and twigs placed on the surface (henceforth referred to as ‘oviposition trays’) was placed in each cage.

Once females were introduced to the cages, oviposition trays were checked twice daily for eggs and once eggs had been laid they were removed to a smaller 0.2 L plastic cup, filled with 60 g of a grass silage:silage water (2:3 mix), and twigs. Once hatched, larvae remained in these ‘holding lagoons’ before being transferred to the neonicotinoid-treated experimental lagoons at five days of age as this was the time when they were large enough to handle (a body length no smaller than 5 mm).

### Pesticide exposure

Neonicotinoid-treated experimental lagoons were created by thoroughly mixing together sawdust solids and grass silage in a 4:1, ratio (hereafter referred to as ‘substrate’). Sixty grams of the substrate was then added to 0.2 L plastic cups (hereafter referred to as ‘lagoons,’ E. Rotheray, 2015, personal communication) and each placed in a tie-top plastic freezer bag surrounded by dried leaves which had been sieved to remove smaller pieces of detritus (Fig. S1).

In order to contaminate the larval growth substrate, a mixture of silage water (700 mL) and bottled water (1 L) (ASDA, own brand) was contaminated to six different levels with analytical grade thiamethoxam using stock solutions (Sigma-Aldrich, Gillingham, UK): 0 (control), 5, 15, 50, 100 and 500 ppb as a positive control (Schaafsma et al., 2015). One hundred and fifty millilitres of each treatment solution was added to each treatment lagoon and stirred thoroughly with a small stick which was left in the lagoon. Five-day old larvae (from date of hatching), were removed from the holding lagoons, gently rinsed in bottled water, blotted dry with paper towel and weighed with a 0.001 g resolution balance (Precisa 125A; Newport Pagnell, Buckinghamshire, UK) before being placed into the treatment lagoons.

Larvae were randomly assigned to treatment groups with 10 individual replicates per treatment group (60 larvae in total per full experiment). Larvae were exposed to thiamethoxam from the day they were introduced to the treatment lagoon, to the day they started to pupate. The full experiment was repeated four times (240 larvae), and each separate experiment was populated with eggs from a different female, to ensure that any genetic variation in tolerance to thiamethoxam did not confound the experimental design (Hemingway et al., 2004). Lagoons contained sticks to allow larvae to climb out to pupate, but were covered with a plastic bag to prevent larvae from escaping. The dried leaves acted as a pupation site. Throughout the experiment, lagoons were kept in a dark room (21 °C) to prevent light degradation of thiamethoxam helping to ensure that there was an equal distribution of thiamethoxam through the lagoon profile (Peña, Rodríguez-Liébana & Mingorance, 2011).

### Larval development

Following Rotheray, Goulson & Bussiere (2016), larval growth was monitored by increase in mass. Every three days the larvae were removed from the treatment lagoons, gently rinsed in mineral water (ASDA, own brand) and blotted dry before being weighed and replaced in the lagoon. If the larvae could not be located, the bag of leaves was searched for larvae or pupae. To ensure there was no degradation of the thiamethoxam, all measurements took place under red light. If a larva was found that had exited the lagoon prematurely and was not pupating, the replicate was removed from the experiment. Pupal mass and date of pupation (±three days) were also recorded. Once pupation had commenced, remaining non-pupating replicates were checked for pupation twice daily. Pupae were weighed on a 0.001 g resolution balance, and individually placed in labelled 50 mL tubes with netting secured over the opening, with a small amount of tissue paper to absorb any excess moisture. These tubes were stored in the dark at 21 °C and five days after pupation were checked twice daily for emergence.

### Adult measurements

Upon emergence, adults were colour-marked on their thorax denoting their treatment group with a spot of non-toxic enamel paint, released into a flight cage (60 cm × 45 cm × 60 cm), and provided with pollen, water, and a 15% sucrose solution for one week. To observe and compare the behaviour of individual flies, seven-day old adults were individually placed into a smaller cage of the same design (30 cm × 20 cm × 25 cm), provided with water and 15% sucrose solution in feeders and a small amount of pollen. They were given 1 min to acclimatise. Using an instantaneous sampling technique (following similar protocols in Gilbert, 1985), behaviour was then recorded for 10 min. These behavioural activity budgets were categorised as: stationary, grooming, walking, flying, probing through the cage netting with their proboscis, feeding on nectar, pollen or water (grouped together as feeding) and moving which involved remaining stationary whilst making small jerking motions of their body.

### Statistical analysis

All statistical analyses were carried out using SPSS (v. 21; IBM SPSS Inc. Armonk, NY, USA). Data from the four experiment replicates were pooled for all analyses. The significance threshold was set at 0.05.

#### Larval development

Data were tested for normality using the Shapiro–Wilk statistic and visual inspection of Q–Q plots, and homogeneity of variance was tested using Levene’s statistic. A one-way ANOVA was used to determine the effect of thiamethoxam on pupal weight. Due to deviations from normality a Kruskal–Wallis *H*-test was used to investigate the effect of treatment on larval development time (five-day old larvae to pupation). Log-transformed larval weight data was compared between treatment groups using a generalised linear mixed model (GLMM) with treatment (thiamethoxam presence or control) and time (day 3, 6, 9 or 12) as fixed factors, ‘experiment round’ (1, 2, 3 or 4) was included as the random effect, and ‘scaled identity’ for the repeated measures covariance structure. We first fitted a full model and systematically omitted interaction terms if they did not increase model fit. Model fit was compared using the Akaike Information Criterion (AIC). AIC was also used in selecting the repeated covariance type in models with repeated measures structure. Fisher’s exact test (2 × 6) was used to analyse the distribution of count data between treatment type and the likelihood to exit a lagoon prematurely or remain in lagoon.

#### Survival analysis

Larvae that reached the pupal stage were counted as survivors, irrespective of whether they later successfully completed metamorphosis (Haider, Dorn & Müller, 2013). Survival of the larvae across the treatment groups was analysed using Kaplan–Meier survival analysis, and the log-rank test with a Bonferroni correction was applied to test for differences between survival distributions across treatment groups. Replicates where larvae were found in the leaves but were not pupating were completely removed from the experiment. Once individuals reached pupation they were treated as ‘censored data’ (i.e. the number of larvae reaching pupation). Censored data across treatment groups was dissimilar and are therefore reported (Table 1). Median lethal concentration (LC_50_) was calculated by probit regression analysis.

**Table 1:** Larval survival, development time and average pupal weight from six different larval populations reared in substrate contaminated with thiamethoxam.

Treatment group	Number of larvae that reach pupation (total *n* of group)	Survival (%)	Average pupal weight (g) ± SD
Control (A)	30 (36)	83.3	0.249 ± 0.0049
5 ppb (A)	27 (36)	75	0.240 ± 0.0056
15 ppb (A)	20 (33)	63.6	0.255 ± 0.0086
50 ppb (A)	27 (35)	77.1	0.250 ± 0.0064
100 ppb (A)	27 (35)	77.1	0.247 ± 0.0057
500 ppb (B)	5 (38)	13.2	0.227 ± 0.0129

**Note:**

Treatments sharing the same letter did not differ significantly at *P* < 0.05 (post-hoc test: pairwise log-rank).

#### Adult behaviour

The total amount of time spent carrying out each behaviour was compared between treatment groups. Assumptions of normality were not met for each group of the independent variables as defined by the Shapiro–Wilk statistic and visual inspection of histograms, and so individual non-parametric Kruskal–Wallis *H*-tests were used to investigate the effect of thiamethoxam treatment on adult behaviour.

## Results

### Larval development

Across treatments, 27 larvae exited the lagoons prematurely and were found in the dried leaves. By the end of the experiment, for the control 5 and 50 ppb groups, four larvae (of 40 replicates in that treatment group) had exited prematurely (10%). Most larvae that were found in the leaves were in the 15 ppb group (7/40, 17.5%) with the least in 500 ppb (2/40, 5%); but overall there was no effect of treatment on exiting larvae (Fisher’s exact test, *P* = 0.656). The lower figure for the positive control (500 ppb) is probably due to the elevated mortality levels of larvae in this treatment. These replicates were removed from all further statistical analyses.

There was no significant effect of treatment on development time, which was 9–13 days (Kruskall–Wallis; *H*(5) = 3.367, *P* = 0.644; median for all groups—12 days), and no effect of treatment on pupal weight (one-way ANOVA, *F*_5, 129_ = 1.029, *P* = 0.403). Larval weight did not significantly differ between treatment groups (GLMM; *F*_5, 762_ = 0.326, *P* = 0.897).

### Survival

**Figure 1:**
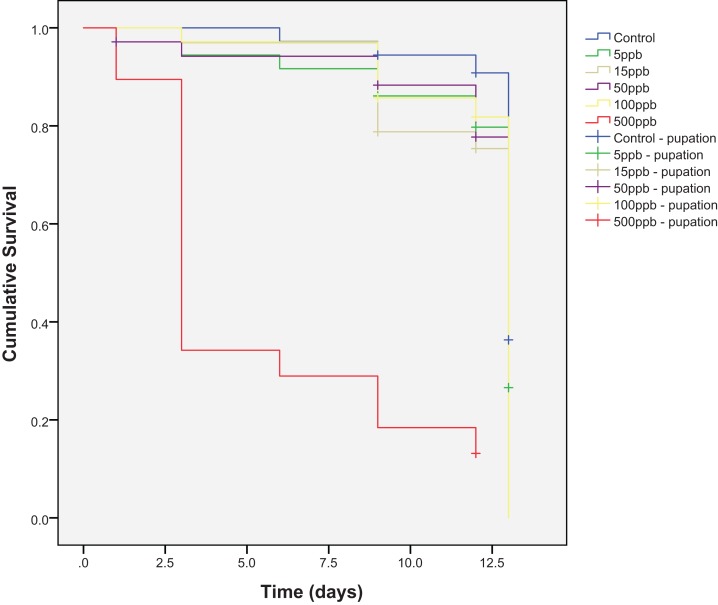
Cumulative survival of *Eristalis tenax* larvae (*N* = 33–38 per treatment) when reared in substrate contaminated with five different concentrations of thiamethoxam, plus control. Crosses indicate individuals that reach pupation (censored data). Many individuals pupated at the same time and so crosses are nested underneath one another. Post-hoc pairwise comparisons (Kaplan–Meier analysis, pairwise log-rank tests) showed significant differences between all groups with 500 ppb.

Mortality across the six treatment groups was significantly different (Kaplan–Meier, log-rank; χ^2^(5) = 122.27, *P* = <0.001) and post-hoc pairwise comparisons showed significant differences between all treatment groups and the 500 ppb group (Kaplan–Meier analysis, pairwise log-rank test: control—500 ppb χ^2^(1) = 50.172, *P* = <0.001; 5–500 ppb, χ^2^(1) = 39.272, *P* = <0.001; 15–500 ppb, χ^2^(1) = 35.431, *P* = <0.001; 50–500 ppb, χ^2^(1) = 36.280, *P* = <0.001; 100–500 ppb, χ^2^(1) = 41.112, *P* = <0.001) (Fig. 1). Percentage survival was lowest in the 500 ppb group (13.2%), and highest in the control (83.3%) (Table 1). The LC_50_ for thiamethoxam and *E. tenax* was 215 ppb.

### Adult behaviour

Distribution shapes were similar for all behaviour groups across treatments as assessed by visual inspection of a box plot. Median scores for all behaviours were not significantly different across treatments (Kruskal–Wallis; time spent: stationary *H*(5) = 4.989, *P* = 0.417; grooming *H*(5) = 8.217, *P* = 0.145; walking *H*(5) = 6.960, *P* = 0.224; flying *H*(5) = 0.980, *P* = 0.964; probing *H*(5) = 3.188, *P* = 0.671; feeding *H*(5) = 7.497, *P* = 0.186; moving *H*(5) = 5.571, *P* = 0.350).

## Discussion

While thiamethoxam has been detected in waterbodies on and near to farmland (Samson-Robert et al., 2014) with the potential for harming non-target species (Pisa et al., 2015; Morrissey et al., 2015) we report little or no effect of larval exposure to field-relevant doses of the neonicotinoid thiamethoxam via contaminated substrate. Our results indicate that *E. tenax* larvae are insensitive to field-realistic doses of thiamethoxam with no significant likelihood of direct mortality, or impacts on growth, development time or activity budgets in the resulting adults. These are the first known published data on the effects of a neonicotinoid on the insect family Syrphidae.

Within the field of aquatic toxicology, the chironomids (Diptera) are widely used in laboratory tests, with most work being undertaken at the organismal level by measuring larval survival and growth (Saraiva et al., 2017). A comprehensive review by Morrissey et al. (2015) looked at the lethal concentration in water (LC_50_) and the EC_50_ values (where 50% of the pesticide’s maximal effect is observed) for 214 acute (24–48 h) and chronic studies (7–28 days) for 48 species of aquatic invertebrate species. The geometric mean taken from the range of the LC[E]_50_s for all Diptera and neonicotinoids tested was 32.9 ppb, and was 9.3 ppb for *Chironomous dilutes* (Diptera: Chironomidae) specifically. Aquatic invertebrate species also appear to vary in their sensitivity with *C. dilutes* being found to be the most sensitive of the three most common aquatic invertebrate species tested (compared to *Daphnia magna* (Cladocera; geometric mean: 23,690 ppb)) and *Gammarus pulex* (Amphipoda; geometric mean: 235.8 ppb)) (Morrissey et al., 2015), which emphasises the importance of testing a wide range of species in addition to a range of chemicals.

From this same review, only two studies examining effects of thiamethoxam on Diptera (Culicidae) were reported: *Aedes aegypti* (24 h) and *Chironomus riparius* (48 h) resulting in an LC[E]_50_ of 183 and 35 ppb, respectively. Thiamethoxam is an order of magnitude less toxic than two other neonicotinoids, imidacloprid and clothianidin, to all life stages of *C. dilutes* over a 14-day exposure. The 14-day median lethal concentrations for imidacloprid, clothianidin and thiamethoxam were 1,520, 2,410 and 23,600 ppb. The 40-day median effect concentrations (emergence) for imidacloprid, clothianidin and thiamethoxam were, 390, 280 and 4,130 ppb, respectively (Cavallaro et al., 2016). Other studies demonstrate that toxicity can differ strongly between closely related species; the chronic LC_50_ of imidacloprid to *Chironomus tentans* is just 0.91 ppb (Stoughton et al., 2008). Unfortunately, a lack of studies on the effects of thiamethoxam on Diptera prevents much in the way of comparison. Our study estimated the LC_50_ for *E. tenax* to be much higher at 215 ppb. It seems possible that thiamethoxam has a generally lower toxicity to aquatic invertebrates when compared to imidacloprid or clothianidin, but clearly more comparative studies are needed to draw firm conclusions.

Earlier larval instars have been consistently shown to be more sensitive to contaminants due to differences in biomass and bioaccumulation after exposure to a contaminant (Heinis, Timmermans & Swain, 1990). Our experiment commenced with five-day old larvae (which was essential to allow handling of larvae), it is possible that if eggs were laid directly in contaminated water, hatching or commencement of growth could be adversely affected.

Despite ensuring the lagoons were not exposed to UV light for the duration of the experiment (as UV is the major component contributing to thiamethoxam’s photolytic decomposition; Gupta, Gajbhiye & Gupta, 2008), it is possible that during the experiment the thiamethoxam degraded over time due to the physicochemical properties of the matrix or bacterial action. Thiamethoxam in contaminated waste water rapidly degrades in darkness and this degradation has been attributed to the presence of microorganisms using the neonicotinoid as an energy source; a lagged effect was noticed as the microorganisms adapted to using the thiamethoxam (Peña, Rodríguez-Liébana & Mingorance, 2011). It is thus possible that the bacterial content of the lagoons resulted in biodegradation of the pesticide. However, if so, we would expect much the same to occur in the field.

Larvae of *E. tenax* mature in stagnant, anaerobic ponds and water-courses where they filter-feed on microbes associated with rotting organic material and faecal matter (Hayes, Levine & Wilson, 2016). It is possible that, due to being adapted to exploit these fetid environments, they are naturally robust and capable of coping with toxins. It is also feasible that their cuticle is impermeable therefore may prevent absorption of the chemical, reducing contact toxicity.

Interestingly, some larvae prematurely exited the lagoon before pupation; some exited just three days after transfer. We found no effect of treatment on the likelihood to exit a lagoon. We therefore hypothesise that larvae may be capable of detecting different conditions, which may be unfavourable compared to those in which they started development. Larvae are known to travel up to 10 m in search of favourable pupation habitats (Fischer et al., 2006), so searching for more favourable larval habitats, or the original habitat from which they were displaced may also be possible.

Evidence from studies on honeybees and bumblebees suggest that there is a latent effect of larval neonicotinoid exposure on the behaviour of the resulting adult. For example, larvae of *Apis cerana* (Apidae) exposed to low doses of imidacloprid (0.24 ng/bee) exhibited significantly impaired olfactory learning when tested as adults (Tan et al., 2015); the same effect was seen in *Apis mellifera* alongside higher brood mortality and reduced adult lifespan (Peng & Yang, 2016). Exposure to thiamethoxam specifically during larval development of the bumblebee can result in decreased memory function (Stanley, Smith & Raine, 2015), and reduced emerging queen body weights, reduced ovary weights, and lowered sperm counts in the honeybee (Gajger, Sakač & Gregorc, 2017). In this study, we found larval exposure to thiamethoxam and its metabolites to have no latent effect on in-situ adult hoverfly activity budgets, though we did not test for effects of high level behaviours such as learning and memory. It is noted that the nervous system of adult insects is very different from that of the larvae, with the structures targeted by neonicotinoids, such as the mushroom-bodies in the brain, being undeveloped in the larvae (Farris et al., 1999). Further work is warranted on adult exposure to pollen and nectar containing field-relevant levels of neonicotinoids, as they pose the same potential risk of harm to hoverflies as they do to bees.

Research is most often focused on the effects of singular chemical exposures. However, fields can be treated with a large number of chemical compounds, with pesticides regularly applied as mixtures of similar or different active ingredients being common practice (Cavallaro et al., 2016; Botías et al., 2017). This potential exposure to a cocktail of chemicals in agricultural run-off is not addressed in this study and has not been commonly addressed in the wider field of investigations on the effect of pesticides on non-target organisms in general. Further research should examine exposure to field-realistic mixtures of chemicals (Rodney, Teed & Moore, 2013).

In summary, we found that thiamethoxam exposure results in elevated mortality of *E. tenax* larvae only at concentrations above those normally found in field-realistic situations. The larvae of this species appears to be less sensitive to thiamethoxam than some other aquatic insects that have previously been examined. Further research is required to investigate possible adverse effects via adult exposure, or from larval exposure to other neonicotinoids and currently used complex mixtures of pesticides. Farmland management may benefit from including hoverfly larval habitat to maintain an important pollinating species which, at least in the larval stage, appears to not be highly susceptible to at least one commonly used pesticide.

## Supplemental Information

10.7717/peerj.4258/supp-1 Supplemental Information 1 Experimental set up. Treatment lagoons were placed inside a large tie-top plastic bag and surrounded by sieved dried leaves to be used as a pupation substrate. The stick allowed larvae to crawl out of the treatment lagoon and pupate.

10.7717/peerj.4258/supp-2 Supplemental Information 2 Larval weight data.

10.7717/peerj.4258/supp-3 Supplemental Information 3 Dataset including development time and survival.

10.7717/peerj.4258/supp-4 Supplemental Information 4 Behavioural observations of the *E. tenax* after larval thiamethoxam exposure. Each line represents one individual and the behaviours recorded in a 10 minute period: (S) stationary, (GR) grooming, (W) walking, (F) flying, (PR) probing through the cage netting with their proboscis, (N) feeding on nectar, pollen or water (grouped together as feeding) and (M) moving which involved remaining stationary whilst making small jerking motions of their body.

## Additional Information and Declarations

### Data Availability

The following information was supplied regarding data availability: The raw data has been provided as Supplemental Dataset Files.
