# Supplementary figures and images for: Effects of chronic exposure to thiamethoxam on larvae of the hoverfly Eristalis tenax (Diptera, Syrphidae)
Source: PeerJ. 2018 Jan 17;6:e4258. doi: 10.7717/peerj.4258 (PMC5775755; doi:10.7717/peerj.4258)

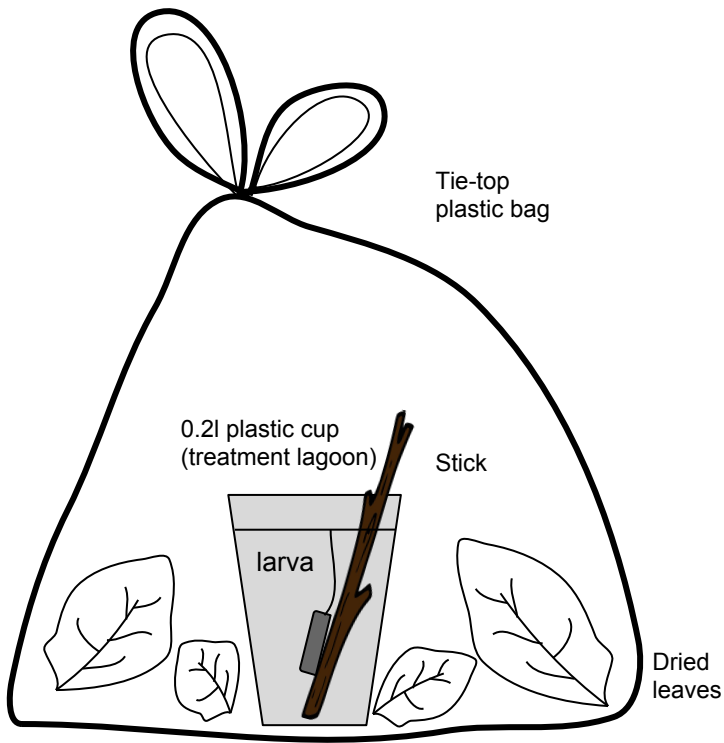

Supplement: Supplemental Information 1 — Treatment lagoons were placed inside a large tie-top plastic bag and surrounded by sieved dried leaves to be used as a pupation substrate. The stick allowed larvae to crawl out of the treatment lagoon and pupate. [file peerj-06-4258-s001.pdf]
